# Characterizing morphological alterations in blood related disorders through Atomic Force Microscopy

**DOI:** 10.7150/ntno.93206

**Published:** 2024-03-25

**Authors:** Rohini Rakshak, Shweta Bhatt, Sushant Sharma, Rutesh Agharkar, Santosh Bodakhe, Rohit Srivastava

**Affiliations:** Department of Bioscience and Bioengineering, Indian Institute of Technology, Bombay, India.

**Keywords:** AFM, Blood related disorders, Cancer, Diabetes, Genetics, Anemia, Pathogens, Diagnosis, Therapy

## Abstract

Atomic Force Microscopy (AFM) is a very flexible method that can create topographical images from a range of materials and image surfaces. Significantly, AFM has emerged as an invaluable tool for dissecting the morphology and biochemical aspects of body cells and tissues. The high-resolution imaging capabilities of AFM enable researchers to discern alterations in cell morphology and understand the underlying mechanisms of diseases. It contributes to understanding disease etiology and progression. In the context of this review, our focus will be directed towards elucidating the pivotal role of AFM in analysis of blood related disorders. Through detailed comparisons with normal cells, we delve into the alterations in size, shape, and surface characteristics induced by conditions such as cancer, diabetes, anaemia, and infections caused by pathogens. In essence, various work described in this article highlights to bridge the gap between traditional microscopy and in-depth analysis of blood-related pathologies, which in turn offers valuable perspectives for both research and clinical applications in the field.

## Introduction

Currently various types of fatal blood related disorders are known, including pathogenic diseases, genetic disorders, diabetes, and certain types of cancers. For the development of therapy for such diseases, accurate diagnosis of the exact condition is essential. Imaging of the cells or tissues is commonly used to measure the size, surface area, and tissue properties 1, which is crucial to diagnose a specific disease or identify the drug design sites against that disease.

To visualize the disease inflicted cells or tissues that are affected in blood diseases, currently numerous imaging techniques are being used. Optical microscopy can be utilized to visualize blood smears, tissue smears or bone marrow smears. However, this microscopy requires the examiner to manually examine the sample, which may result in highly subjective conclusions with poor repeatability 2, 3. Moreover, optical microscopy has its resolution limited to the wavelength of natural light. The maximum resolution achieved by optical microscopy is ~200 nm 4. Similarly, confocal microscopy provides a good signal to noise ratio, but the image processing is extremely time consuming 5. Fluorescence microscopy utilizes fluorescent molecules like Cy5, GFP (green fluorescent protein), or FlAsH (fluorescent arsenical helix binder) that are bound to probes which are then introduced to the cells using tools such as microinjection. These probes then bind to the cells to their target proteins and produce fluorescence when a certain wavelength of light is passed through them 6, 7. Limited penetration, scattering of light due to tissues, photobleaching of dyes, and the limited amount of time the experiment can be run are the major drawbacks of fluorescence microscopy 8. Detailed images of blood related cells or tissues can be provided using scanning electron microscopy (SEM) and transmission electron microscopy (TEM) which has a resolving power of ~5 nm 9. TEM can however only produce two dimensional images black and white images 10. Moreover, the cells need to be preserved in vacuum 3 which makes it unfit for *in vivo* imaging of blood diseases.

Atomic force microscopy (AFM) can prove to be a powerful tool to investigate blood samples. The structural changes in cells or tissues related to diseases can be identified using the AFM 11, 12. The interactions between AFM probe and the surface of the sample produces topographic images. The AFM probe scans the surface of the sample while the cantilever of the probe moves perpendicularly according to the interatomic potentials between the sample and the tip of the cantilever. The cantilever's perpendicular movements dictate the topographic images of the sample surface 13, 14. AFM is advantageous as it allows the imaging of the cells in their natural conditions such as in buffer solutions, *in situ*, and *in vitro*. The sample preparation is easy, and the high signal-to-noise ratio of AFM allows topological imaging with resolution of upto 0.5-1 nm (lateral resolution) and 0.1 - 0.2 nm (vertical resolution) 15-17.

Biochemical assays and spectroscopic tools for biomarker identification are disadvantageous as they tend to have low sensitivity, require large amount of samples, and are time consuming and are expensive 18-21. The structural and compositional changes in the cells or tissues are common characteristics of several diseases which can be utilized as a biomarker by AFM 22-25.

Another added advantage of AFM is the measurement of stiffness and elasticity using Young's modulus (E) 26. For blood cells like red blood cells (RBCs), change in membrane stiffness or elasticity can be identified with the AFM which is otherwise not present in other imaging techniques which makes it an ideal choice for investigating blood related samples 27. Studies have shown that pathological erythrocytes have a higher Young's modulus than normal erythrocytes 28. Sample collection for analysis via AFM is simple and only requires blood samples or tissue surgical specimens from patients, as well as from healthy volunteers for control 29.

AFM also allows the measurement of membrane surface roughness. The surface roughness is commonly measured as root mean squared roughness (R_rms_) or average roughness (R_a_) 30. The decrease in membrane roughness for the likes of erythrocytes might indicate a pathological sample 31-33. The roughness on the cell surface can hint the presence and size of biomolecules like protein or polysaccharides, which is otherwise not possible in light microscopy. The roughness can also indicate the changes of the cell surface structures, which can be combined with other macroscopic observations to diagnose a condition 34, 35.

Diseases such as diabetes, anaemia, malaria, hereditary spherocytosis (HS), and blood cancers which directly affects the erythrocytes, leukocytes and other blood corpuscles have already been studied using AFM where structurally and functionally abnormal blood components were observed. In this review, investigations done by AFM in blood related disorders is highlighted, and the potential for AFM to be used in diagnosis of these diseases in the future is focused.

## Application of AFM in blood cancers

AFM has been used to image tumour cells or tissue, specifically in different blood cancers. AFM examinations of leukocytes in leukemia patients have shown a significant increase in the number of needle-like features on their surface with an increase in surface roughness than that of healthy white blood cells. Additionally, when comparing normal cells to tumour cells from the same source, AFM could identify if the cells are malignant or no and show the notable differences in cell membrane shape between the two types of cells. This can offer a trustworthy supplementary foundation for differential and clinical pathological diagnosis. Similar abnormal observations along with some common types of blood cancer, have been studied and highlighted in this section 36.

The noticeable cytoskeletal changes, in AFM indentation measurements have revealed that cancer cells are typically softer than healthy ones. On the other hand, cancer tissues seem to be considerably stiffer than healthy cells. This is because of an overabundance of extracellular matrix proteins that are stiff, like collagen, fibronectin, and laminin.

The intricate interaction between cells and their extracellular matrix (ECM), which controls biochemical reactions and essential cellular functions, is upset by these alterations to the mechanical equilibrium. AFM is a quantitative technology that has the potential of integration with a traditional histological investigation of surgically removed tissues in this particular situation 37.

### Chronic myeloid leukemia

**Figure 2:**
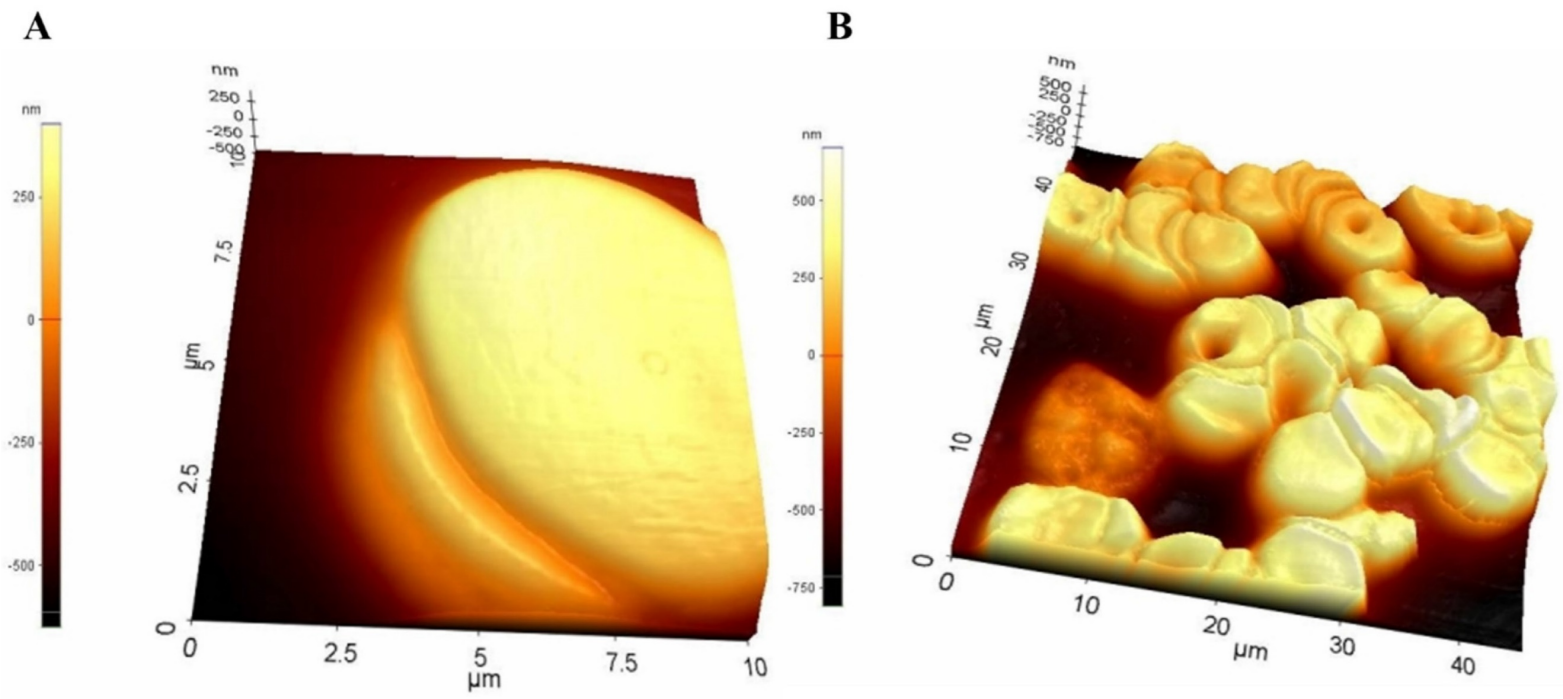
Surface morphology of leukocytes **(A)** from healthy individuals **(B)** from chronic leukaemia patients obtained using AFM. Reproduced with permission from Medical University Publishing House Craiova 2013 38.

Chronic myeloid leukemia (CML) is a type of cancer that starts in certain blood-forming cells of the bone marrow. An abnormal gene called BCR-ABL, turns a normal cell into CML cell. CML is a fairly slow growing leukemia, but it can change into a fast-growing acute leukemia that is hard to treat. A study was conducted on nine patients with chronic myeloid leukemia in varying stages of the disease—three in the chronic phase, three in the accelerated phase, three in the blastic phase. As a control three healthy human subjects had their bone marrow taken. The morphology of the cells was examined using non-contact mode of AFM. The scan resonant frequency was 1.7 kHz with a Z scan range of 25 μm. The height of normal cells was observed within the range of 10-15 micrometres (µm), whereas CML cells were consistently observed showing multiple spicules on the cell surface ranging from 25-30 micrometres (µm) showing a significant difference of roughness between normal cells and leukemic cell (Figure 2) 38.

### Myeloma

**Figure 3:**
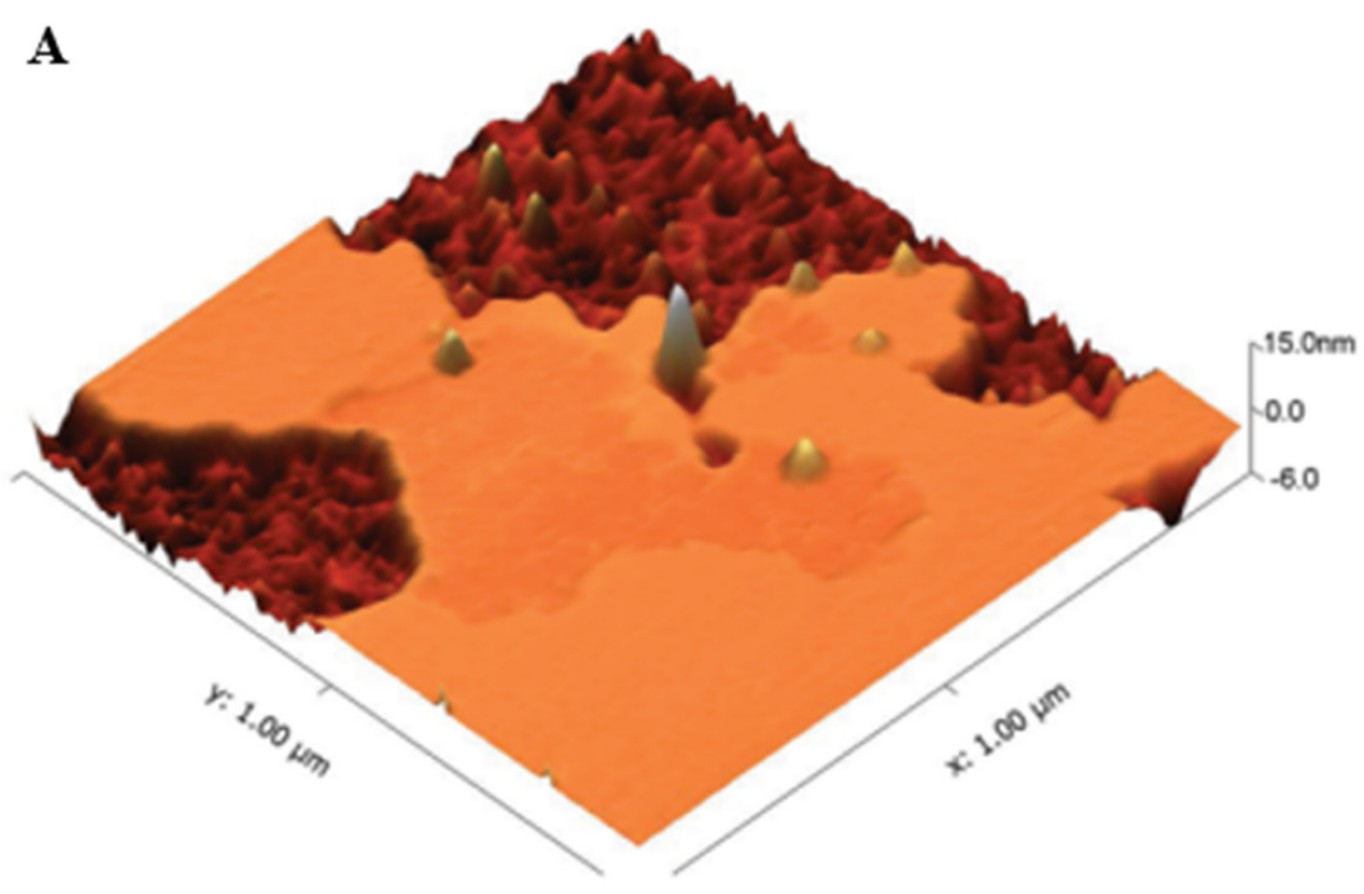
Leukocyte surface from chronic leukemia individual with protruding λFLC visualized using AFM. Reproduced with permission from Biochemical Society 2013 41.

The haematological cancer myeloma or multiple myeloma (MM) is caused by clonal plasma cells in the bone marrow. Free immunoglobulin light chains (FLCs), are secreted by MM plasma cells and are a predictive indicator of illness 39, 40. λFLCs (lambda free immunoglobulin chains) is one such type of FLC which was thoroughly studied using the AFM in MM cells. In the study, to visualise λFLCs, 30 μg/ml of λFLC was put into recently broken mica and left for 30 minutes. The mica was then washed with TBS. After the mica surface was cleaned, the sample was added to 250 μg/ml λFLC or a protein-free control, and it was left to incubate for one to two hours. Using AFM, the sample was imaged in a liquid cell using tapping mode. An E-type scanner with a maximum scan area of 12.5 μm^2^ and a vertical range of 3.4 μm was employed for the imaging process (Figure 3) 41. In the study, it was concluded that at the cell surfaces of multiple myeloma aggregated alpha light chains which were due to overexpression of genes typical in cancer. Furthermore, these chains were interacting zwitter-ionically with transport molecules across the cell membrane. These transport molecules were acting as recognition sites for cargo delivery, enhancing cancer cell life 41.

In another study the extracellular vesicles from bone marrow cells, were studied from two groups of patients: healthy and multiple myeloma. It was observed that, based on Young's moduli values, multiple myeloma cells were significantly smaller than healthy cells. The multiple myeloma altered the structure of these vesicles, making them elastic from their healthy counterparts. These elastic bodies would be assisting myelomas to flourish within patient body 42.

AFM studies of similar manner also paved way for novel formulations like Rituzimab, Truxima, Ruxience, and Rixathon for therapeutic application for multiple myeloma and lymphoma 43.

## AFM based studies on Diabetes Induced Alterations

Type 2 diabetes mellitus (T2DM) disrupts architecture and functionality of numerous bio-physiological elements at a molecular scale. Diabetes being a complex disorder has driven researchers to explore its impact at atomic-level. In this section, we delve into significant studies made possible through AFM in elucidating the intricate morphological changes within body cells, tissues and fluids associated with diabetes.

**Figure 4:**
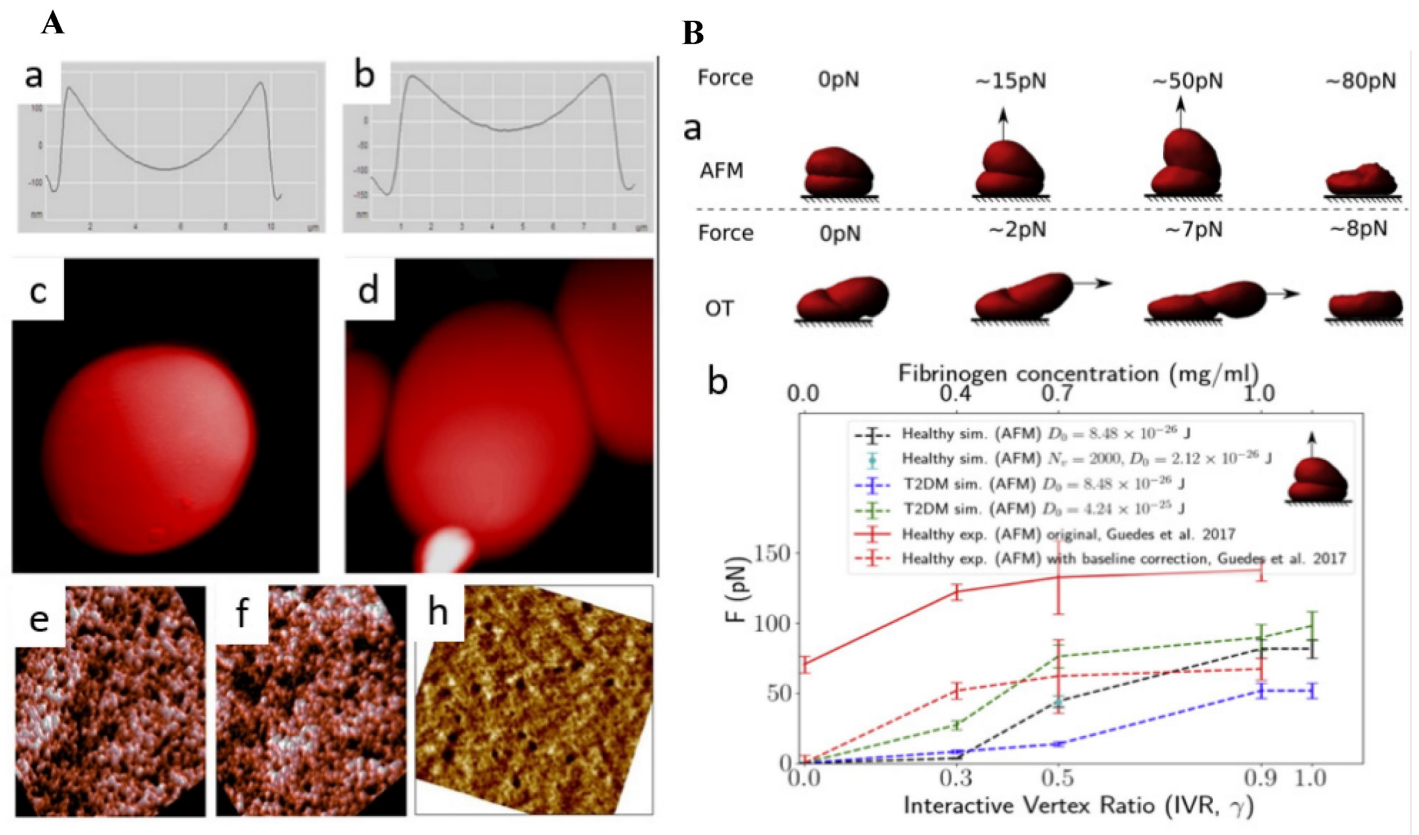
(A) Geometrical characteristics of (a) healthy RBC and (b) pre-diabetic erythrocyte. Distinct, balloon-like structure of the RBC of the diabetic patient (c) clinging and (d) adhering of cells among themselves. Erythrocyte cell membrane AFM images of (e) healthy (f) pre-diabetic and (h) diabetic (Scan size is 900 × 900 nm). Reproduced with permission from MDPI 2018 45. (B) (a) Simulations of increasing force exerted on the top of healthy doublet (b) critical force positively correlated with interactive vertex ratio. This corresponds to the fibrinogen concentration level. Reproduced with permission from Cell Press 2020 46.

Using AFM, researchers were able to examine subtle changes in the morphology of erythrocytes, such as their height, axial ratio, concave depth, and thickness. A study revealed how individuals with prediabetes and diabetes exhibit alterations in erythrocyte morphology and how glycated hemoglobin (HbA1c) might have a significant role in altered morphology. 44. AlSalhi et al. revealed the presence of subtle surface irregularities in the erythrocytes of individuals with prediabetes and diabetes, such as mild pits and blowholes. Notably, diabetic patients exhibited a balloon-like structure in the RBCs. Another significant finding was the tendency of these cells to adhere to one another, forming clusters (Figure 4A) 45. Fibrinogen, a key glycoprotein in the clustering of RBCs, is naturally occurring process that has significant implications on rheology of blood and blood flow. It plays crucial role in type 2 diabetes mellitus (T2DM). Deng et al., used AFM to quantify, fibrinogen-dependent clumping of RBCs in T2DM patients by a predictive computerized simulator. To begin simulations, one RBC was placed on top of another to make a doublet. The top RBC was gradually dragged away from the bottom RBC until the doublet separated completely. The applied force was then correlated with the concentration of fibrinogen (Figure 4B) 46.

Alexandrova et al. investigated the morphological parameters of human leukocytes in T2DM using the tapping mode. In comparison to the healthy donors, the diameter of leukocytes and Young's modulus was found to be increased significantly, although cell height reduced. Thus, white blood cells (WBCs) from diabetic patients were found to be stiffer than leukocytes from healthy donors. A number of spherical prominences and depressions changed significantly indicating influence of diabetes WBCs malformation 47. Another study also found a similar change in WBC stiffness. Young's modulus was found to be raised by 73% in diabetes patients, which is roughly 3.7 times greater than in healthy individuals, as demonstrated by AFM scans of WBC cytolemma surfaces 48.

In addition to erythrocytes and leukocytes, AFM has been employed to investigate a range of tissues, bodily fluids, and other constituents of blood. The findings of these investigations have been summarized in Table 1, covering several key parameters derived from the study of these biological elements.

## AFM in anemia

AFM has been utilized to investigate the morphological alterations in blood corpuscles, specifically erythrocytes, caused due to genetic conditions. Some of these well-studied cases recorded in the literature are discussed in this section.

### Genetic-alteration based anemia

#### Sickle cell anemia

Unexpectedly low amount of erythrocyte or hemoglobin in the blood is referred to as anemia. Erythrocyte shape disfigurement is another common observation in anemic conditions 55. Sickle cell anemia is a type of anemia that is caused because of point mutation in β-globin gene. It is a recessively inherited gene and causes the production of abnormal (sickle shaped) hemoglobin 56-58, which in turn results in malformed RBC production-sickle shaped instead of normal biconcave. Sickle shaped RBCs (SSRBCs) have lower flexibility, high adhesiveness to the endothelium, as well as other blood corpuscles (RBCs, leukocytes and platelets), and an increased viscosity than wild type (WT) RBCs 56, 59-63.

In a study, determination of the stiffness of anomalous erythrocytes was done using AFM with blood samples collected from known sickle cell anemia patients 64. Comparison of the Young's modulus obtained from the anemic erythrocytes was done against the Young's modulus of the erythrocytes of healthy patients. Young's modulus of the anemic RBCs was 3.05 ±1.09 kPa, which was significantly higher than the normal cells whose Young's modulus was 1.10 ± 0.40 kPa. Young's modulus is a direct measure of stiffness. The increase in erythrocyte stiffness in sickle cell anemia is hypothesized to be due to the affinity of actin filament with spectrin filament which is caused by the abnormal hemoglobin HbS.

#### Thalassemia

Thalassemia is a genetic condition where the shape of the erythrocytes is disfigured due to the abnormal α or β chain of hemoglobin, which in turn causes cytoskeletal changes 65. Thalassemia is phenotypically similar to some types of anemia like iron deficiency anemia (IDA). Thus, a proper distinction between the two types of diseases becomes extremely vital for their treatment.

In a study 66, AFM was employed to distinguish the length, width, length-width ratio, valley, peak, valley-peak ratio, standard deviation and surface fluctuation of normal healthy erythrocytes, IDA erythrocytes, and THAL (thalassemic erythrocytes). AFM topographic data of the erythrocytes showed a distinction between all three types*.* The ratio of length-width for IDA was found to be 1.55 ± 0.42 which signified a more oval cell shape, whereas compared to THAL whose length-width ratio was 1.06 ± 0.34. Another important observation was the size of the membrane particles. Healthy cells had the membrane particles of 8 nm whereas anemic cells had the membrane particle size of 140 nm. This suggested that membrane proteins aggregated on the erythrocyte surface. The THAL on the other hand had holes and creviced ultrastructures on the cell surface strongly suggesting the damage of membrane skeleton of erythrocytes 67.

#### Glucose-6-phosphate-dehydrogenase (G6PD) deficiency anemia

Defects in the gene coding for glucose-6-phosphate-dehydrogenase (G6PD) is a hereditary disorder which can cause defective G6PD or complete absence of G6PD. This in turn results in hemolytic anemia. These anemias have a wide range of symptoms ranging from being extremely severe, to being asymptomatic 68, 69

In a study 70, AFM was employed in a tapping mode to obtain the ultrastructures of G6PD deficient erythrocyte and normal erythrocytes. The cantilever had thickness, width and length of 0.3, 32, and 30 µm respectively, along with 400 kHz oscillation frequency and 4 N/m of constant force. Imaging of the cells were done in air at 256 x 256 pixels and a 1.95 Hz scan speed. 33 different G6PD deficient individuals were chosen for the test and on genetic testing, 9 types of mutations were observed-7 missense, 1 same sense, and 1 intron variant. Using statistical tools incorporated with AFM, a difference in roughness of the membrane was observed in membrane of all types of mutants. G6PD deficient erythrocytes were significantly rough than the normal erythrocytes.

Hereditary Spherocytosis (HS) refers to a disorder where the erythrocytes are spherical instead of the normal biconcave. The symptoms of this disorder include anemia, splenomegaly, jaundice, hemolysis, and even growth retardation. Mutations of genes coding for proteins of the erythrocyte membrane as well as cytoskeletal proteins such as α- and β-spectrin, ankyrin or protein 4.1 cause hereditary spherocytosis 71. This causes the membrane to take up the lowest volume structure-a sphere 72. Diagnosis of this disorder is normally done by flow cytometry, light microscopy or electron microscopy 73. However, the major limitation of these methods is the lack of measurements in the *z-*axis. AFM can be utilized to overcome this challenge 74.

**Figure 5:**
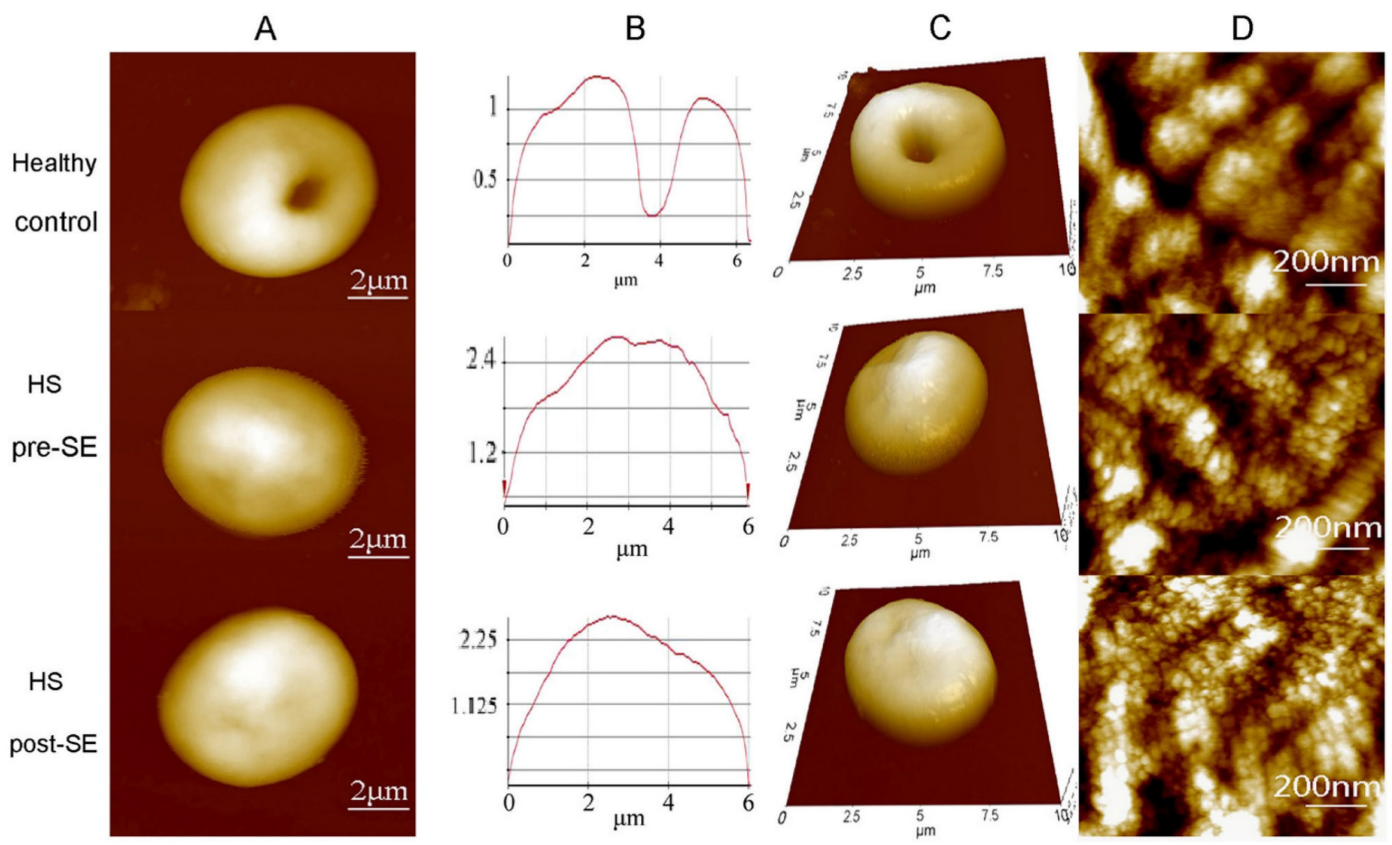
Compared AFM topographic images from healthy individuals, HS individuals pre-splenectomy, and HS individuals post-splenectomy. **(A)** single erythrocytes, **(B)** height profile of the same erythrocyte **(C)** 3D model of the same erythrocyte **(D)** Surface ultrastructures of the corresponding erythrocytes. Scan area: (**A, B,** and **C)** 10 µm x 10 µm; **(D)** 1 µm x 1 µm**.** Reproduced with permission from Springer 2016 75.

A study conducted utilized tapping mode of AFM in air to obtain the topographic images of the RBC samples 75. The topographic images obtained for the samples showed a distinctive spherical structure of the erythrocytes. The surface roughness (Ra) was significantly reduced in the HS erythrocytes (215.67 ± 6.11 nm) than in the normal erythrocytes (287.00 ± 44.03 nm). The patients underwent splenectomy after the initial test and their erythrocytes were re-examined after 3 months using AFM. There was no change in the shape of erythrocytes shape after splenectomy, however there was a slight increase in their sizes. The compared AFM images are given in Figure 5.

In another study 76, AFM was employed as well to discriminate normal erythrocytes from HS erythrocytes. Lateral force maps were obtained by quantitating the torsion value of the cantilever and then representing them in arbitrary units. These lateral force maps were then used to measure the median sliding force (F_f_) and root mean square roughness (R_q_). The AFM probes enabled force spectroscopy, with which the Young's modulus was obtained. The Young's modulus and average friction forces were ~ 20% higher in the HS surface regions than in the surface of the discocytes.

#### Hemochromatosis

Transferrin receptor 2 (TfR2) is a counterpart of the conventional transferrin receptor 1 (TfR1). TfR2 exists in two forms, namely, α and β isoforms. TfR2α, which binds diferric transferrin (Tf), is implicated in cellular iron metabolism. Mutations in TfR2 are associated with hemochromatosis, indicating its role in regulating iron levels. This interaction was confirmed using AFM. TfRα, located on the cell surface, acts as an essential iron donator by binding to transferrin (Tf) and facilitating the delivery of iron to the cells 77.

### Nutritional deficiency-based anemia

#### Folate deficiency

A study revealed how deficiency of folate is one of the main causes of megaloblastic anemia (MA) 78. Through AFM it was found that the erythrocytes of the patients exhibited greater size compared to those of the healthy controls, yet their altitude, average roughness, Rp-v and surface area was lower in comparison to the controls 79.

#### Iron deficiency anemia

Iron deficiency anemia (IDA) is a worldwide prevailing condition caused due to the reduced intake of iron through diet, bleeding due to injury, loss of blood due to menstruation, or gastrointestinal bleeding 80. In a study AFM was employed to obtain the topographic images of IDA erythrocytes 81. In comparison to the RBCs of healthy patient and even thalassemic RBCs, the IDA erythrocytes showed higher deformities and irregularities in morphology. The cells were oval and more swelling was observed at the centre of the RBCs.

## AFM in blood diseases due to pathogens

The AFM technology has the potential to be used for screening newly emerging pathogens. It is useful for pathogen research since it offers a quick, easy, and secure method for characterizing viruses with minimal and comparatively unrefined preparation. Several blood diseases caused by microbes that have been studied using AFM are listed in this section.

### Dengue

Dengue is a mosquito-borne flavivirus that poses a significant threat to global public health 82. Platelet dysfunction and thrombocytopenia are significant hematopathologic characteristics of dengue fever 83, 84. A study utilized tapping and contact mode of AFM to investigate the impact of viral infections on platelets. Morphological changes were observed in human blood platelets following exposure to the dengue-2 virus. The study concluded that exposure to dengue-2 virus at concentrations similar to those found in naturally occurring viremic stages in human infections can stimulate platelets by increasing their production of P-selectin and their ability to bind fibrinogen 85.

### COVID-19

**Figure 6:**
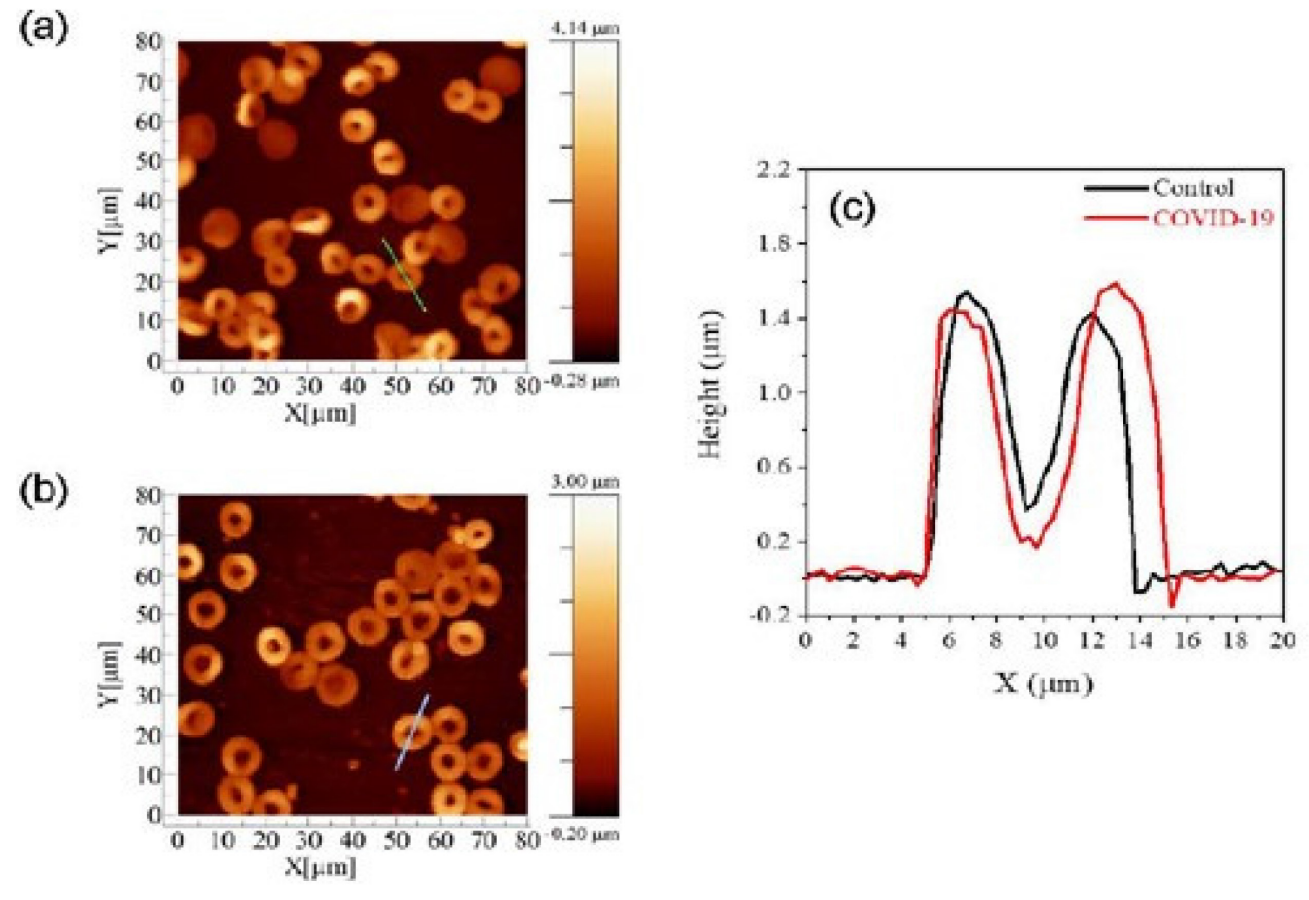
AFM images of erythrocytes of (a) control and (b) COVID-19 affected cells. (c) Distribution of the cells concerning the parameter of diameter and height for both diameter and height. (d) Box plots of the diameter of cell. Reproduced with permission from SciELO 2023 87.

Human erythrocytes are harmed structurally, functionally, and morphologically by SARS-CoV-2 86. In order to examine changes in structural and biomechanical features by AFM, tapping mode was employed to image erythrocytes from control and COVID-19 affected groups in a study. Utilizing a typical AFM setup with a combined optical microscope, the cellular morphology images and elasticity data were acquired. This combination allowed AFM to be positioned laterally and tip over the cell's nuclear area with micrometer-scale accuracy. The acquired images are given in Figure 6. Young's modulus of COVID-19-affected-RBCs were than normal RBCs which the authors hypothesize to be due to membrane alterations that cause the membranes to stiffen. The average RBC measurements for control volunteers were 8.92 ± 0.73 μm for diameter and 1.27 ± 0.11 μm for height. In contrast, the RBC measurements for COVID-19 patients showed 10.41 ± 0.78 μm for diameter and 0.98 ± 0.07 μm for height 87.

### Malaria

One of the major problems during malaria infection is the attachment of infected RBCs (iRBCs) to the arterioles, capillaries and venules, collectively called as microvasculature walls. A study developed an advanced flow-based adhesion assay to demonstrate the adhesion of iRBCs to endothelial CD36 receptor protein. Adhesion receptor and ligand binding kinetics were analyzed using AFM 88. AFM was also used to investigate how knobs arise and how they interact with the spectrin network of the host cell. *P. falciparum* erythrocyte membrane protein 1 (PfEMP1) and knob associated histidine-rich protein (KHARP) are two examples of parasite-expressed proteins that are exported by the parasites to the host cell membrane during the maturation process. These proteins formed knobs on the host cell surface, stiffen the membrane, and induce cytoadherence, or cell stickiness. Both the extracellular and cytoplasmic surfaces of infected erythrocyte membranes were examined using AFM. The cytoplasmic surface AFM pictures revealed additional information about the spectrin network. On the cytoplasmic surface of infected erythrocytes, knobs and their links or connections to the spectrin network were readily visible. Knobs seen from the cytoplasmic surface resembled those seen from the extracellular surface in terms of size and distribution. Moreover, significant alterations in the spectrin network were observed subsequent to infection; that is, a partial disruption of the spectrin network during the schizont stage 89.

### HIV

Hu, M. et al, recognized that CD4+ T cells are essential for the immune regulation of numerous viral diseases, including HIV-1. This study describes the use of AFM-based single-molecule force spectroscopy to measure the morphological changes and particular binding forces between the CD4 antibody and its receptor. One CD4+ T cell that was activated and one resting cell were used for the measurements. Topographic images were obtained using a contact mode. For force detection, AFM-based force spectroscopy was employed. Conventional retraction was used to create force-distance curves. Every force-distance curve experiment was carried out at an identical loading rate 90.

Exosomes are external membrane vesicles (EV) at the nanoscale that are important in signalling and communication between cells. These are a potentially useful technique for drug delivery in biomedicine. Himbert S. et al, investigated the biomechanical characteristics of both kinds of particles in liquid and air using the atomic force microscope technology. Bruker's Bioscope Catalyst and Bioscope Resolve microscopes in PeakForce QNM mode were used to do the measurements in both liquid and air 91.

**Figure 7:**
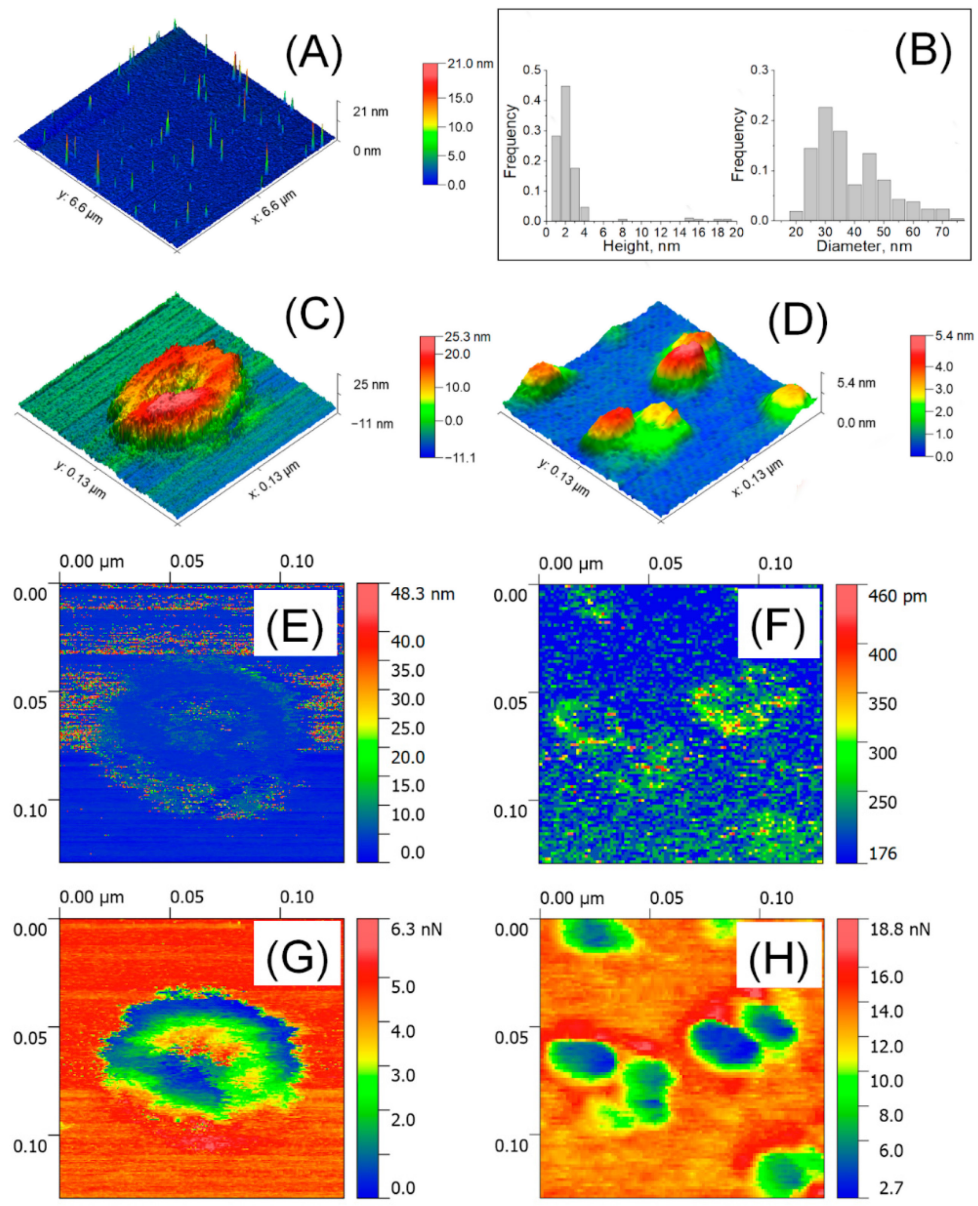
Detection of EV by AFM in air. **(A)** 6.6 × 6.6 μm^2^ large scaled AFM image. **(B)** Distribution of size;** (C)** High resolution AFM image of a single exosome (129 × 129 nm^2^) and **(D)** single exomeres; **(E, F)** Deformation; **(G, H)** adhesion forces. Reproduced with permission from MDPI 2020 90.

**Figure 8:**
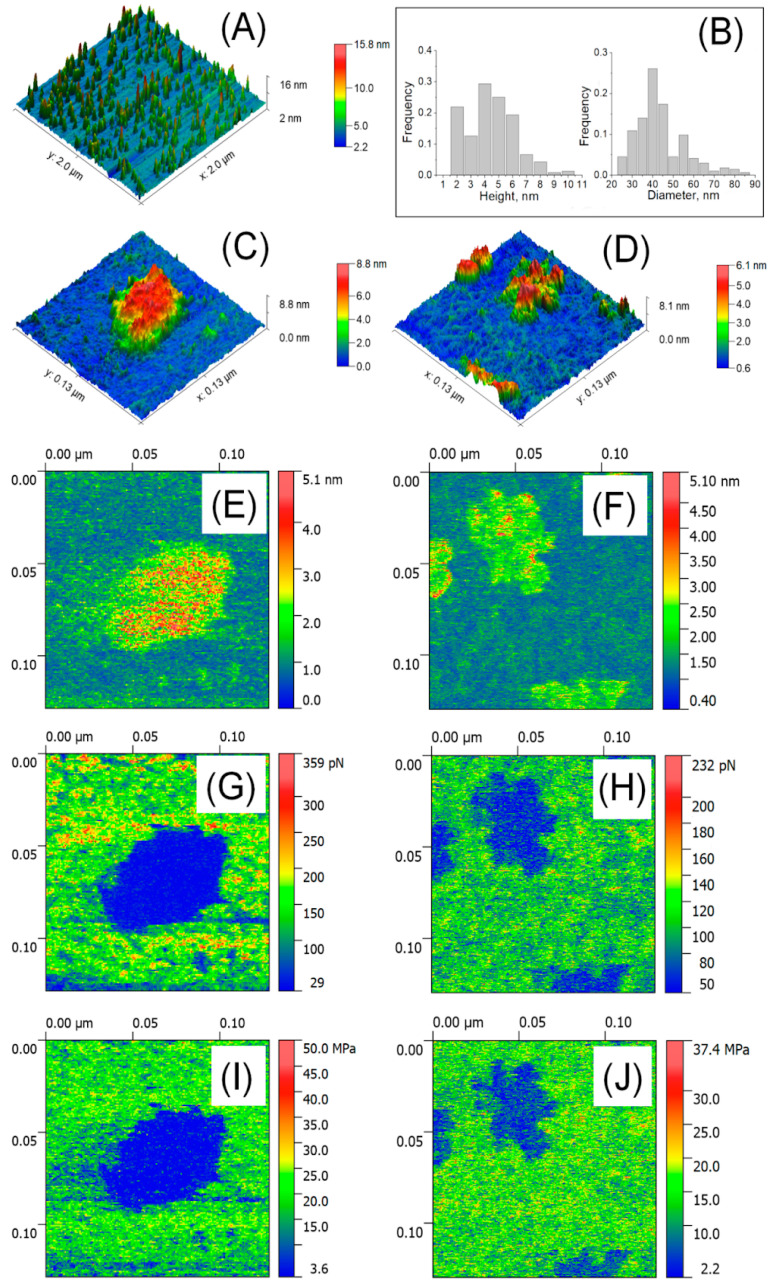
Detection of EVs in liquid using AFM. **(A)** 2 x 2 µm^2^ image; **(B)** Distribution of size (n=207-254); **(C)** High resolution image of a single exosome (129 x 129 nm^2^); **(D)** Aggregates of single exomere; **(E, F)** Parameters of deformation; **(G, H)** Forces of adhesion; **(I, J)** Young's modulus. Reproduced with permission from MDPI 2020 90.

Atomic force microscopy (AFM) was used to analyze isolated blood plasma vesicles at the single particle level in both liquid and air. These vesicles carried biomarkers linked to exosomes and exomeres. Exosome tests in the air showed the presence of highly sticky spots within a mechanically depressed interior cavity. On the other hand, the particles' measured diameter was about 35 nm, and the highly sticky exomere sites were situated in their periphery. The internal chamber of exosomes exhibited reversible deformation in liquid state, and a slightly distorted lipid bi-layer was detected. On the other hand, exomeres had an observed diameter of around 16 nm and were not distorted. A stronger sorption of water film in the air could be linked to the differences in diameters (Figure 7, Figure 8) 92.

## Conclusion

Diseases alter the morphology and mechanical properties of cellular and non-cellular components of affected tissues. The ability of AFM to distinguish diseased blood tissues is promising as highlighted in this review. Rapid technological developments in single-molecule force spectroscopy may reveal the precise mechanisms of intermolecular behaviours in such diseases. AFM developments have the potential to assist pathologists in the diagnosis and grading of tissue biopsies or whole blood samples by precise qualitative observations.

The identification of biochemical and nanomechanical signals will be advantageous with the use of AFM in conjunction with other spectroscopic imaging methods. These combinations offer prognostic indicators for the disease as well as targets for treatment that allow for the analysis of the biochemical makeup and mechanical characteristics (such as stiffness or flexibility) as the disease progresses. AFM also provides promising imaging outcomes in cell surface receptors, host-pathogen interaction, cell-cell interactions, and drug-cell interactions.

In addition to imaging, force-distance curves produced using AFM have a great deal of promise for clarifying drug discovery, infectious diseases, therapeutic approaches, and the creation of novel diagnostics. It is feasible to investigate a variety of interactions, from molecules to cells, by altering the AFM tip.

The functionalization of tips opens up the range of systems that can be studied providing access to previously unattainable fast dynamic processes like ligand-induced oligomerization of receptors and transporters, the conformational dynamics of transporters, receptors, and channels during transport, signalling, and gating. This review provides a fresh view of AFM as a high-technology research tool for future applications.

The combination of AFM with imaging, force mapping, and spectroscopic techniques will prove, beneficial in understanding and management of diseases and disorders.

## Figures and Tables

**Figure 1:**
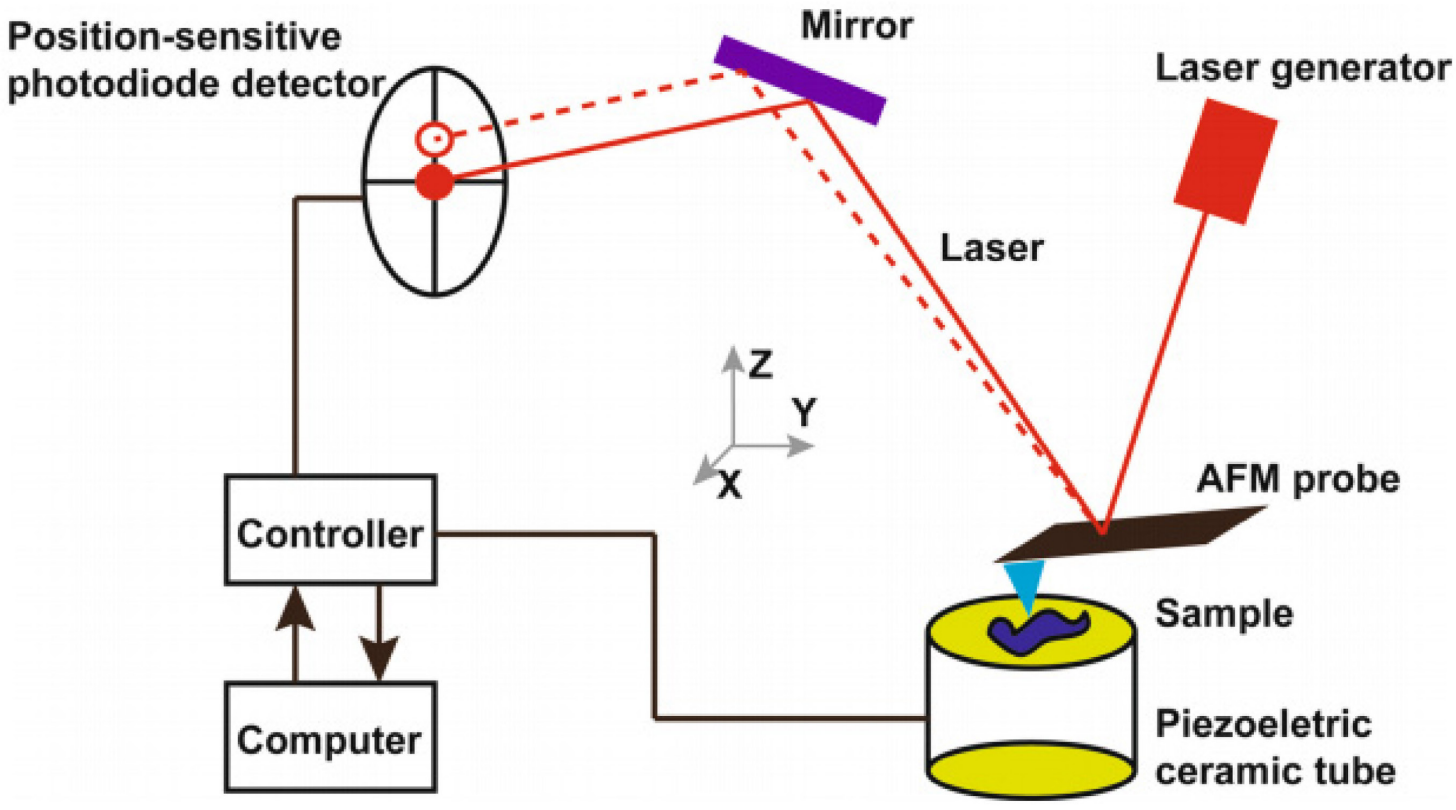
Working principle of atomic force microscopy. Reproduced with permission from Springer 2018 36.

**Table 1:** A summary of studies utilizing Atomic Force Microscopy (AFM) to investigate the effects of diabetes on different types of body cells and various bodily fluids.

Type	Physiological Element	AFM Technique used	Sample preparation	Morphological Parameters	Specifications/Observations **	References
Cell components	Erythrocyte	Tapping Mode Cantilever thickness: 7 μm, Length: 225 μm, width 38 μm, Resonance frequency: 190 kHz, Force constant: 48 N/m, Al-coating	Erythrocytes pellet was fixed in glutaraldehyde (2.5 % in H2O), and washed 3X with 0.2 M PBS, a drop was added on silicon oxide (300 nm). Air dried	**Pre-diabetic**		44
				Height	-0.80 μm	
				Axial ratio	-0.09 μm	
				**Diabetes Mellitus**		
				Height	-0.46 μm	
				Concave depth	-0.29 μm	
				Axial ratio	+0.08 μm	
				Thickness	+0.32 μm	
	Erythrocyte	Spring constant: 20-80 N/m. Tip radius 8 nm Resonant frequency: 342-394 KHz	Erythrocytes mixed with Acetone (Spectroscopic grade)	**Pre-diabetic**		45
				Diameter	-1.26 μm	
				Concave depth	-154.1 nm	
				Roughness	-0.02 nm	
				**Diabetic**		
				Diameter	+0.89 μm	
				Concave depth	Concave	
				Roughness	+0.11	
	Erythrocyte	RBCs were resuspended in autologous plasma after blood was centrifuged, resulting in a 2-2.3% hematocrit.	The force required for cell-cell separation was measured by placing the AFM tip above the RBC core, which creates an upward pull in a vertical direction. Vertex fraction, Force required was calculated.	Critical Force (F) Vertex Ratio	F was found to be higher than healthy ones. F is favourably related to the level of fibrinogen concentration and increases with the interactive vertex ratio g.	46
	Leukocyte	Tapping mode with scanning probe microscope (scan frequency 0.6-0.8 Hz), tip radius curvature: 10 nm	Leukocyte ring collected after centrifuge was re-suspended in RPMI-1640	T2DM		47
				Diameter	+7.5%	
				Height	+13.1%	
				Young's Modulus (E)	+23.7%	
				Globular prominences and depressions	+6.1%	
	Leukocyte	Contact Mode in aqueous solution. Scanning performed: silicon probe Stiffness: 0.06 N/m radius curvature: 10 nm.	Separated lymphocytes cultured in Hank's medium for 20 min. Medium changed to: Medium 199 for 60 min	Insulin-dependent		48
				Diameter	+0.3 μm	
				Height	-0.36 μm	
				Young's Modulus	+0.41 MPa	
				Adhesion	+3.51 nN	
				Roughness (Sa)	-39.96 nm	
				Non-insulin dependent		
				Diameter	-1.79 μm	
				Height	-0.18 μm	
				Young's Modulus	+0.38 MPa	
				Adhesion	-1.32 nN	
				Roughness	-48.14 nm	
	Cardiomyocytes	Tapping Mode Tip radius: 2nm	TyBS (1.8 mM Ca2+ or 200 μM Ca2+) treated cells plated on polylysine-coated glass micro slide chambers (10 min). Attached cells + Propidium Iodide (5 min, dark), 3X washes: TyBS with different ionic compositions	Height	+0.7%	49
				Apparent Elastic Modulus	+112%	
				Adhesive Force	+10.5 X	
Tissue	Adipose	Contacting and intending k ~ 0.035 N/m frequency: 17 kHz tip radius: 10 nm	Subcutaneous and visceral fat were extracted from the abdomen wall and the larger omentum. These were divided into pieces and placed on coverslips coated with polylysine.	Mean Elastic Modulus	+7.02 kPa	50
Other body components	Ocular basement membranes. Tip Radius: 6 nm	Nominal Spring constant 0.081 N/m	Inner limiting membranes (ILM) coated with 10 μg/ml polylysine	Thickness of ILM	Increased	51
				Stiffness	Increased	
	Achilles Tendon	Both contact and tapping modes Atrractive force of 0.01 nN Tip radius: 15 nm	Sample collection from *Rattus norvegicus*.	Coronal Plane Area Dimesions	+3.76 mm^2^	52
				Size of Transverse section area	+0.388 mm^2^	
Body fluids	Tear fluid	Tapping mode Radius of curvature: 10 nm	Tear samples collected by flushing method using saline solution.	Dendrites (on 1-year treatment with insulin and anti-diabetic drug)	Dense network observed.	53
				Surface roughness	19 nm	
	Clot formations	Tip radius: 8 nm Resonance frequency: 75 kHz Spring constant: 3 N/m	Peripheral blood was obtained. 2% CaCl_2_ added to initiate coagulation.	Morphology:		54
				Erythrocyte	Poikilocytosis Anisocytosis	
				Leukocyte	Irregular surface with extended pseudopodia	

** - (Minus)/+ (Plus): Decrease/Increase observed in parameter with respect to normal cell.

**Table 2:** A summary of studies utilizing Atomic Force Microscopy (AFM) to investigate the effects of anemia on erythrocytes.

Disorder studied using AFM	Physiological Element	AFM technique used	Sample preparation	Morphological parameters	Specifications/Observations	References
Sickle cell anemia	Erythrocyte	Non-contact; scan rate 0.2 Hz; probes with 0.01 N/m spring constant.	AFM grade mica was coated with poly-L-lysine; 5.0% RBCs in PBS were allowed to adhere to the mica. Fixation of cells was done using 0.5% glutaraldehyde, followed by washing with PBS.	Young's modulus	+1.95 kPa	64
Thalassemia	Erythrocyte	Contact mode; 255 kHz oscillation frequency; 0.03 N/m force constant; 2.5 N/m spring constant.	RBCs were fixed using 2.5% paraformaldehyde, and diluted in PBS. Sample was air dried.	Length-to-width ratio	+0.04	67
				Peak-valley distance	-749.14 nm	
				Ra	-213.58 nm	
G6PD deficiency anemia	Erythrocyte	Tapping mode; 400 kHz oscillation frequency; 4 N/m force constant; 1.95 Hz scan speed.	RBCs were washed with PBS and them deposited on mica. Using a fast stream of PBS through a needle at 20° angle, RBCs were sheared open and the membrane was obtained.	Ra	Increased	70
Hereditary spherocytosis	Erythrocyte	Tapping mode; 2.5 N/m spring constant.	Blood was centrifuged and the pellet was resuspended in PBS. The suspension was fixed on a coverslip with 1% glutaraldehyde, followed by washing using deionized water.	Length	-0.59 µm	75
				Width	-0.41 µm	
				Peak-valley distance	-214.5 nm	
Hereditary spherocytosis	Erythrocyte	0.6-0.7 Hz scanning rate.	RBC resuspension was fixed using 1% glutaraldehyde.	Relative friction force	+0.19	76
				Relative RMS roughness	-0.2	
				Young's Modulus	+0.19	
Folate Deficiency	Erythrocyte	Contact mode, tip diameter 20 nm, coefficient of tip 2.5 N/m	Blood was withdrawn from patients with MA. Erythrocytes were separated and fixed on a glass slide.	Size	+	79
				Altitude	-	
				Average roughness	-	
				Rp-v	-	
				Surface area	-	
Iron Deficiency	Erythrocyte	Contact mode; 287-336 kHz oscillation frequency; 28-45 N/m force constant.	Cells were fixed using 2.5% paraformaldehyde.	Topographical information	Crenated Shape Smaller size of RBC Large holes (abnormal hemoglobin)	81

** - (Minus)/+ (Plus): Decrease/Increase observed in parameter with respect to normal cell.

**Table 3:** A summary of studies utilizing Atomic Force Microscopy (AFM) to investigate the effects of pathogens on blood components.

Disease	Physiological Elements	AFM Technique used	Sample preparation	Morphological Parameter	Specification/Observation	References
Dengue	Platelets	Tapping mode, contact mode	Blood platelets were washed and then exposed to dengue-2-virus before being imaged	Surface changes	Filopodia-like extension and micropitting present	85
COVID-19	Erythrocytes	Tapping mode, force-curve based imaging mode	Muscovite mica sheets were adhered to a glass slide, coated with 0.1% poly-L-lysine for 10 mins at 20 °C, rinsed with 3 ml of buffer A (10 mM Tris-HCl pH 7.5 and 100 mM NaCl), and then dried using an N_2_ flux.	Diameter	10.41 ± 0.78 µm	87
				Height	0.98 ± 0.07 μm	
				Average RBC measurement		
				Diameter	8.92 ± 0.73 μm	
				Height	1.27 ± 0.11 μm	
HIV	Extracellular vesicles	Contact mode	For air measurements: 10 µl of the investigated solution was diluted 100 times. Following a five-minute incubation period, it was washed with deionized water and dried.	Diameter	35nm	92
			For liquid measurements: Poly-L-lysine solution was applied to the newly cleaved mica. Mica was dried in N_2_ stream with 60 µl of the investigated solution after 5 mins of incubation. The probe was soaked in the drop that covered the mica surface after 5 mins of incubation.	Diameter	16nm	

## Abbreviations

AFM: Atomic Force Microscopy
RBC: red blood cell
R_rms_: root mean squared roughness
R_a_: average roughness
CML: chronic myeloid leukemia
MM: multiple myeloma
FLC: free immunoglobulin light chain
λFLC: λ free immunoglobulin light chain
T2DM: type 2 diabetes mellitus
WBCs: white blood cells
IDA: iron deficiency anemia
THAL: thalassemic erythrocytes
G6PD: glucose-6-hosphate dehydrogenase
HS: hereditary spherocytosis
EV: external membrane vesicles
